# Genome-wide methylation profiling reveals differentially methylated genes in blood DNA of small-cell lung cancer patients

**DOI:** 10.1093/pcmedi/pbac017

**Published:** 2022-06-22

**Authors:** Yanqi He, Calvin Wei, Zhifu Sun, Julie M Cunningham, Liang Wang, Zong Wei, Ping Yang

**Affiliations:** Division of Epidemiology, Department of Quantitative Health Sciences, Mayo Clinic, Scottsdale, AZ 85259, USA; Department of Respiratory and Critical Care Medicine, West China Hospital, Sichuan University, Chengdu 610041, China; Department of Physiology and Biomedical Engineering, Mayo Clinic, Scottsdale, AZ 85259, USA; School of Molecular Science, Arizona State University, Tempe, AZ 85281, USA; Division of Computational Biology, Department of Quantitative Health Sciences, Mayo Clinic, Rochester, MN 55905, USA; Genomic Shared Resource, Mayo Clinic Cancer Center, Rochester, MN 55905, USA; Program of Tumor Biology, Moffitt Cancer Center, Tampa, FL 33612, USA; Department of Physiology and Biomedical Engineering, Mayo Clinic, Scottsdale, AZ 85259, USA; Division of Epidemiology, Department of Quantitative Health Sciences, Mayo Clinic, Scottsdale, AZ 85259, USA

**Dear Editor**,

Small-cell lung cancer (SCLC), one of the most-deadly malignant tumors, accounts for ∼15% of all lung cancers.^1,2^ Although highly responsive to the standard chemoradiotherapy, the recurrence rate of SCLC is nearly unity, with median survival time ranging from 2–6 months. Almost all SCLC patients are inoperable, leading to the difficulty in obtaining adequate biopsy tissue for research.^3^ Consequently, there is an urgent need to find noninvasive biomarkers to predict the risk and provide early diagnosis of SCLC. We aimed to identify potential epigenomic markers for SCLC development in blood DNA and reveal differentially methylated genes through a multi-omics approach, from genome-wide DNA methylation profiling, followed by pathway enrichment analyses and target gene validation utilizing Gene Expression Omnibus (GEO) and Oncomine microarray databases, to TCGA Pan-Cancer cohorts. It is known that virtually all patients with SCLC are victims of tobacco smoking,^4^ but it is unknown whether stopping smoking before SCLC diagnosis would influence the epigenetic biomarkers under investigation. We here report a study that identified aberrantly methylated genes between current and former smokers among SCLC patients, revealing a set of candidate biomarkers in peripheral blood DNA for better stratifying patients with high risk.

Specifically, we analyzed 47 paired SCLC cases and controls with a pathological diagnosis of primary tumor at the Mayo Clinic (USA), under an Institution Review Board (IRB) approved protocol (225–99). Enrolment, diagnosis, and data collection processes are provided in the online supplementary material. By a matching design, no difference in age, sex or pack-year was found between cases and controls (Table S1, see online supplementary material). A total of 27 patients (57.4%) had limited-stage and 20 (42.6%) had extensive-stage SCLC. All subjects smoked cigarettes; >50% of cases (59.6%) and controls (53.2%) being current smokers at the time of diagnosis, with mean pack-years of 44.6 and 44.3, respectively.

***Differentially methylated CpG sites in SCLC*.** The magnitude of methylation of 25 550 CpG were analyzed between cases and controls (Fig. 1A), resulting in 46 differential CpG sites between cases and controls (37 hyper- and 9 hypo-methylated). The distribution of differentially methylated CpGs showed that >60% were located outside of CpG islands (CGIs) [Fig. S1 (see online supplementary material), 16 inside and 30 outside of CGIs]. For the 16 In-CGIs, the overall methylation status was significantly higher in cases than in controls (β = 0.58 ± 0.19 vs 0.53 ± 0.18, *P* < 0.001). For the 30 Out-CGIs, the overall methylation status remained higher in cases than in controls (β = 0.52 ± 0.18 vs 0.50 ± 0.17, *P* < 0.001). The Manhattan plot indicates that the 46 SCLC CpGs are spread across all autosomes (Fig. S2, see online supplementary material).

**Figure 1.**
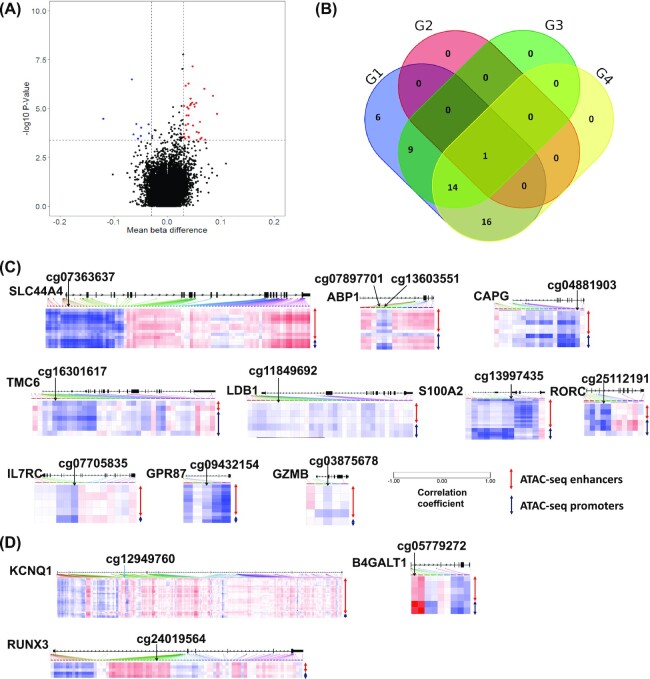
Identifying differential methylation sites in SCLC. (**A**) Volcano plot of the different methylation levels. The red points in the plot represent the upregulated deffrecially expressed genes (DEGs). The blue points in the plot represent the downregulated DEGs. In total, 94 of the sites showed false discovery rate (FDR) < 0.1, and 46 of these CpGs showed a different DNA methylation β value (>0.03) between the SCLC and control groups. (**B**) Venn diagram of the 46 differentially methylated CpGs stratified by smoking status. G1: all subjects group: comparing all cases and all controls; G2: control group: comparing current smokers and former smokers in controls; G3: former smoker group: comparing cases and controls who were former smokers; G4: current smoker group: comparing cases and controls who are current smokers. (**C**) List of CpGs for which the methylation level is negatively correlated with candidate *cis*-regulatory elements (cCRE, including all putative promoters and enhancers) accessibility. For each gene, the heatmap shows the correlation coefficient between the methylation of an individual CpG and cCRE accessibility. CpGs identified in this study are labeled with arrows. (**D**) List of CpGs for which the methylation is positively correlated with cCRE accessibility.

***Differentially methylated CpGs stratified by smoking status*.** To further classify the differentially methylated CpGs stratified by smoking status, we conducted four comparisons (Fig. 1B): all subjects (G1), controls (G2), former smokers (G3), and current smokers (G4). As a result, 154 CpGs showed significant differences in all subjects, 141 CpGs showed significant differences in the controls, 550 CpGs showed significant differences in former smokers, and 93 CpGs showed significant differences in current smoker. All 46 CpGs in the overall analysis were identified as significantly differentially methylated CpGs in the subgroup analysis. As shown in Fig. 1B, the distribution of the 46 SCLC CpGs among the 4 comparisons was: 6 CpGs (4 hyper- and 2 hypo-) were found in all subjects (G1), 9 CpGs (8 hyper- and 1 hypo-) in former smokers (G3), 16 CpGs (12 hyper- and 4 hypo-) in current smokers (G4), 14 CpGs (13 hyper- and 1 hypo-) in both former and current smokers (G3 and G4), and 1 CpG with hypermethylation was identified in all groups (G1–G4).

***Pathway analysis of genes with differentially methylated CpGs*.** These 46 differential CpGs were mapped to 43 genes (Table S2, see online supplementary material). A total of 16 genes with aberrantly methylated CpGs indicated a high risk for SCLC in current smokers, while 9 aberrantly methylated genes indicated a high risk in former smokers. Fourteen methylated genes were determined to increase the risk of SCLC in both former and current smokers. Six methylated genes being identified only in the SCLC group suggested that they were hazardous to SCLC regardless of smoking status. One methylated gene was observed in all four comparison groups, indicating that it is a risk factor for smokers independent of SCLC. KEGG pathway analysis (Fig. S3, see online supplementary material) showed that the 43 genes were involved in apoptosis, inflammatory bowel disease, and hypoxia-inducible factor (HIF)-1 signaling. The 16 differentially expressed genes related to SCLC in current smokers were related to inflammatory bowel disease, adipocytokine signaling, and hematopoietic cell lineage. The 9 genes related to SCLC in former smokers were mainly enriched in drug metabolism. The 14 genes associated with SCLC in both former and current smokers were mainly enriched in transcriptional misregulation in cancer and apoptosis.

***Validation of genes indicated by differentially methylated CpGs using GEO datasets*.** To evaluate the reproducibility, we analyzed the methylation profile in 28 SCLC tissue samples and 13 noncancerous lung tissues from GSE50412. Of the significantly differentially methylated CpGs found in our study, only one aberrant methylation (cg04992673) of the *HEPACAM2* gene showed a significant difference between SCLC and adjacent normal tissues in GSE50412. To further examine the biological relevance, we examined the gene expression using GSE43346. Eight differentially methylated genes showed differential expression (FDR < 0.01) in lung cancer: (up: *SOD3, CBX7, RORC, ABHD14A, NDUFV1, LGALS*, and *PLD4*; down: *MPHOSPH9*). In the subgroup analyses, *SOD3, CBX7*, and *RORC* genes were associated with susceptibility of SCLC in current smokers, while the *ABHD14A* gene was associated with susceptibility in former smokers; *MPHOSPH9* and *NDUFV1* genes were strongly associated with SCLC risk in both current and former smokers; and LGALS and PLD4 genes showed a high risk to the development of SCLC but independent of smoking status.

***Oncomine analysis*.** To further confirm these findings, we identified 5 genes with significant dysregulation in lung cancer based on Oncomine microarray databases. The *MPHOSPH9* gene showed significant upregulation in SCLC.^5,6^ The *PLD4*,^7^*ABHD14A*,^7^*NDUFV1*,^7–9^ and *RORC*^8^ genes showed significant upregulation in non-SCLC rather than SCLC.

***Correlation between differentially methylated CpGs and chromatin accessibility in the PanCancer cohort**.* To interrogate whether the methylation of CpGs identified in this study is dynamically regulated in human cancer and correlates with cancer-specific changes of chromatin accessibility, we utilized the Pan-Cancer cohort, which contains 404 samples with both genome-wide DNA methylation and chromatin accessibility profiles.^10^ We found that cg07363637 (*SLC44A4*), cg07897701/cg13603551 (*ABP1*), cg04881903 (*CAPG*), cg03875678 (*GZMB*), cg16301617(*TMC6*), cg11849692 (*LDB1*), cg13997435 (*S100A2*), cg25112191 (*RORC*), cg07705835 (*IL7RC*), and cg09432154 (*GPR87*) were negatively correlated, while cg1249760 (*KCNQ1*), cg05779272 (*B4GATLT1*), and cg24019564 (*RUNX3*) were positively correlated with the accessibility of promoters and/or enhancers (Fig. 1C,D).

In summary, our findings indicate that methylation and genome profiles are significantly different between SCLC patients who continue smoking and those who have quitted before diagnosis, suggesting that the blood-based DNA methylated CpGs could be a potential marker for the early detection of SCLC, with differing candidates based on patients' smoking status.A potential mechanism of genes discovered in our study is the relevance to immune response in SCLC; particularly, some of the enriched Gene Ontology (GO) categories point to inflammation and even hematopoietic lineages. Therefore, we speculate that the changes in DNA methylation may correlate to, and/or be a result of changes in the immune component. Delineation of this mechanistic link will be the focus of future studies to investigate whether the differential methylation and methylation-indicated genes before vs. after smoking cessation constitute feasibly testable markers for early diagnosis of SCLC.

## Acknowledgements

We acknowledge the support from Mayo Clinic Foundation and U.S. National Cancer Institute (Grants No. R03-77118, R01-80127, and R01-84354).

## Supplementary Material

pbac017_Supplemental_File

## References

[1] Burki TK. Treatment options not taken for non-small-cell lung cancer. Lancet Oncol. 2017;18(3):e135. 10.1016/S1470-2045(17)30066-9. 28139407

[2] Torre LA , Bray F, Siegel RL et al. Global cancer statistics, 2015. CA Cancer J Clin. 2015;65(2):87–108.. 10.3322/caac.21262. 25651787

[3] Anderson WC , Boyd MB, Aguilar J et al. Initiation and characterization of small cell lung cancer patient-derived xenografts from ultrasound-guided transbronchial needle aspirates. PLoS One. 2015;10(5):e0125255. 10.1371/journal.pone.0125255. 25955027 PMC4425530

[4] Alexandrov LB , Ju YS, Haase K et al. Mutational signatures associated with tobacco smoking in human cancer. Science. 2016;354(6312):618–22.. 10.1126/science.aag0299. 27811275 PMC6141049

[5] Bhattacharjee A , Richards WG, Staunton J et al. Classification of human lung carcinomas by mRNA expression profiling reveals distinct adenocarcinoma subclasses. Proc Natl Acad Sci. 2001;98(24):13790–5.. 10.1073/pnas.191502998. 11707567 PMC61120

[6] Garber ME , Troyanskaya OG, Schluens K et al. Diversity of gene expression in adenocarcinoma of the lung. Proc Natl Acad Sci. 2001;98(24):13784–9.. 10.1073/pnas.241500798. 11707590 PMC61119

[7] Okayama H , Kohno T, Ishii Y et al. Identification of genes upregulated in ALK-positive and EGFR/KRAS/ALK-negative lung adenocarcinomas. Cancer Res. 2012;72(1):100–11.. 10.1158/0008-5472.CAN-11-1403. 22080568

[8] Su LJ , Chang CW, Wu YC et al. Selection of DDX5 as a novel internal control for Q-RT-PCR from microarray data using a block bootstrap re-sampling scheme. BMC Genomics. 2007;8:1, 140. . 17540040 10.1186/1471-2164-8-140 PMC1894975

[9] Landi MT , Dracheva T, Rotunno M et al. Gene expression signature of cigarette smoking and its role in lung adenocarcinoma development and survival. PLoS One. 2008;3(2):e1651. 10.1371/journal.pone.0001651. 18297132 PMC2249927

[10] Corces MR , Granja JM, Shams S et al. The chromatin accessibility landscape of primary human cancers. Science. 2018;362(6413):eaav1898. 10.1126/science.aav1898. PMC6408149 30361341

