# Supplementary material for: Genome-wide methylation profiling reveals differentially methylated genes in blood DNA of small-cell lung cancer patients
Source: Precis Clin Med. 2022 Jun 22;5(3):pbac017. doi: 10.1093/pcmedi/pbac017 (PMC9306013; doi:10.1093/pcmedi/pbac017)
Supplement: pbac017_Supplemental_File [file pbac017_supplemental_file.docx]

**Supplemental Materials**

**Study Design and Methods**

**Study cohort and data collection.** All patients with a pathological diagnosis of primary SCLC were evaluated and treated at Mayo Clinic (Rochester, MN). A detailed explanation of the enrollment, diagnosis, and data collection process has been reported previously.^1-3^ Briefly, newly diagnosed SCLC was identified by a daily electronic pathology reporting system. Once identified, patients provided consent and were enrolled. A full medical record abstraction was carried out to obtain demographics, the history of tobacco exposure, pack-year, lung cancer pathologic type and clinic stage. All controls were selected from community residents, identified by having had a general medical examination and a remaining blood sample from routine clinical tests. Current smokers were defined as those who smoked at least one cigarette a day within 12 months prior to the blood draw. Former smokers were those who had ever smoked at least one cigarette a day but had quit smoking more than 12 months prior to the blood draw. We excluded individuals diagnosed with major organ failure (e.g., brain, heart, lung, kidney, or liver) on or prior to their visit. To minimize the heterogeneity, we restricted subjects to those of European descent (Caucasian). The Mayo Clinic Institutional Review Board approved this project.

**DNA extraction.** Genomic DNA was extracted from 5 ml whole blood following manufacturer protocol from Qiagen (Germantown, MD USA). For bisulfite conversion, we used the EZ DNA Methylation Kit from Zymo (Orange, CA) to treat 1 μg genomic DNA.^4^ Samples were stored at -20°C and downstream profiling were performed in two weeks.

**DNA methylation analysis.** To identify differential methylation sites between SCLC patients and controls, we analyzed previously tested (unpublished) results using Infinium Human-Methylation27 BeadChip from Illumina. The methylation percentage (β value) at individual CpG sites were calculated using BeadStudio (Illumina). The β value represents proportion of the methylated alleles in all alleles (both methylated and unmethylated CpG), ranging from 0 (no methylation) to 1 (completely methylated).

**Differential methylation analysis.** As described previously, MethLAB was used to determine the significantly differentially methylated CpGs.^5^ False discovery rate (FDR) correction (Benjamini-Hochberg) was used to determine the statistical significance.^6^ Differentially methylation were defined as D_β_ ≥0.3; FDR <0.1.

**Pathway analysis.** To identify enriched gene categories,, we used DAVID (<http://david.abcc.ncifcrf.gov>) to identify KEGG categories.^7^ The pathway network was visualized by Cytoscape.^8^ A Bonferroni *P* value < 0.05 was used.

**Validation using GEO datasets.** To validate our findings, two datasets from the GEO database were downloaded. The methylation profiling microarray of GSE50412 contains 28 fresh frozen SCLC samples and 13 noncancerous lung biopsies. The expression profiling microarray of GSE43346 contains 23 SCLC samples and 42 normal lung tissue biopsies. Analyses were performed by R.

**Oncomine analysis.** To further validate the functional relevance of differential methylated genes in lung cancer, we extracted the gene expression from Oncomine ([www.oncomine.org](http://www.oncomine.org)).^9^ The gene expression differences between cancerous and matched normal lung tissues were then determined. The statistical significance was defined by *P* <0.05 (student t-test).

**Correlation between CpG and chromatin accessibility**. To interrogate the correlation between CpG and the chromatin accessibility of cCREs, we extract ATAC-seq and Illumina Human methylation 450 datasets from GDC Pan-Cancer (PanCan) cohort (<https://xenabrowser.net/>). For each differential methylated CpG associated gene, we extract all CpG and cCRE(defined by PanCan as enhancers and promoters) values and calculate correlation coefficient between each CpG-cCRE pair.

**Statistical analyses** include Chi-square tests to compare demographic characteristics and subset analysis for smoking status. Cellular heterogeneity was adjusted according to a previous algorithm.^10,11^

**Supplemental Tables**

**Table S1. Clinicopathological characteristics of 47 paired SCLC case and control**

| Variables | Cases |  | Controls | Total | P value |
| --- | --- | --- | --- | --- | --- |
|  | (n=47) |  | (n=47) | (n=94) |  |
| Age |  |  |  |  | 0.7619 |
| Mean (SD) | 65.6 (6.5) |  | 65.9 (5.8) | 65.8 (6.2) |  |
| Median | 66.0 |  | 66.0 | 66.0 |  |
| Q1, Q3 | 61.0, 69.0 |  | 62.0, 70.0 | 61.0, 69.0 |  |
| Range | (54.0-81.0) |  | (57.0-78.0) | (54.0-81.0) |  |
| Gender |  |  |  |  | 0.8310 |
| Female | 17 (36.2%) |  | 18 (38.3%) | 35 (37.2%) |  |
| Male | 30 (63.8%) |  | 29 (61.7%) | 59 (62.8%) |  |
| Smoking status |  |  |  |  |  |
| Former | 19 (40.4%) |  | 22 (46.8%) | 41 (43.6%) |  |
| Current | 28 (59.6%) |  | 25 (53.2%) | 53 (56.4%) |  |
| Pack-year |  |  |  |  | 0.9216 |
| Mean (SD) | 44.6 (7.4) |  | 44.3 (8.5) | 44.5 (7.9) |  |
| Median | 45.0 |  | 44.0 | 44.5 |  |
| Q1, Q3 | 40.0, 48.0 |  | 40.0, 50.0 | 40.0, 49.0 |  |
| Range | (22.0-63.0) |  | (15.0-63.0) | (15.0-63.0) |  |
| Stage |  |  |  |  |  |
| Missing | 0 |  | 47 | 47 | - |
| Limited | 27 (57.4%) |  | 0 (0.0%) | 27 (57.4%) |  |
| Extensive | 20 (42.6%) |  | 0 (0.0%) | 20 (42.6%) |  |
| Grade |  |  |  |  |  |
| Missing | 0 |  | 47 | 47 | - |
| III | 2 (4.3%) |  | 0 (0.0%) | 2 (4.3%) |  |
| IV | 35 (74.5%) |  | 0 (0.0%) | 35 (74.5%) |  |
| Not gradable | 10 (21.3%) |  | 0 (0.0%) | 1. 21.3%) |  |

**Table S2. The description of subset groups**

| Group and definition | | Description of analysis | Covariates considered |
| --- | --- | --- | --- |
| G1 | All subjects | Comparing all cases and all controls | Adjusted by sex, age, smoking status, pack-years, and cellular heterogeneity |
| G2 | control group | Comparing current smokers and former smokers in controls | Adjusted by sex, age, pack-years, and and cellular heterogeneity |
| G3 | former smoker group | comparing cases and controls who were former smokers | Adjusted by sex, age, pack-years, and cellular heterogeneity |
| G4 | current smoker group | comparing cases and controls who are current smokers | Adjusted by sex, age, pack-years, and cellular heterogeneity |

| **Table S3. The distribution of 46 differentially methylated CpGs in the subgroup analysis** | | | | |
| --- | --- | --- | --- | --- |
| Group | CpG | Symbol gene | CpG island | Methylation |
| G1 | cg19853760 | LGALS1 | False | hypo |
|  | cg27394566 | PLD4 | False | hyper |
|  | cg07363637 | SLC44A4 | True | hyper |
|  | cg08418332 | CCL19 | False | hyper |
|  | cg25511807 | MMP7 | False | hypo |
|  | cg10467098 | Bles03 (C11orf68) | False | hyper |
| G1 G2 G3 G4 | cg23580000 | ADCY7 | True | hyper |
|  | cg26417554 | RPUSD3 | TRUE | hyper |
|  | cg13603551 | ABP1 | FALSE | hyper |
|  | cg07897701 | ABP1 | FALSE | hyper |
|  | cg17250929 | S100A5 | FALSE | hypo |
|  | cg13424229 | CPA3 | FALSE | hyper |
|  | cg09701102 | NDUFV1 | False | hyper |
|  | cg20981615 | TXK | False | hyper |
|  | cg16522484 | C14orf49 (SYNE3) | False | hyper |
|  | cg18328933 | ABHD14A | True | hyper |
|  | cg04992673 | LOC253012 (HEPACAM2) | FALSE | hyper |
| G1 G3 G4 | cg04881903 | CAPG | False | hyper |
|  | cg03875678 | GZMB | False | hypo |
|  | cg12949760 | KCNQ1 | True | hyper |
|  | cg07732037 | MPHOSPH9 | False | hyper |
|  | cg03311899 | GPR109A | True | hyper |
|  | cg05779272 | B4GALT1 | True | hyper |
|  | cg09076584 | FLJ25006 | False | hyper |
|  | cg24019564 | RUNX3 | True | hyper |
|  | cg09450238 | BTBD6 | True | hyper |
|  | cg16301617 | TMC6 | False | hyper |
|  | cg27625732 | TBC1D13 | True | hyper |
|  | cg11849692 | LDB1 | False | hyper |
|  | cg15551881 | TRAF1 | False | hyper |
|  | cg02593766 | EPN3 | False | hyper |
| G1 G4 | cg16098726 | GP9 | True | hyper |
|  | cg13997435 | S100A2 | False | hypo |
|  | cg25112191 | RORC | False | hyper |
|  | cg07109801 | C3orf60 | True | hyper |
|  | cg07705835 | IL17RC | False | hyper |
|  | cg00983520 | CPT1B | True | hyper |
|  | cg18042806 | MGC15875 | True | hypo |
|  | cg09432154 | GPR87 | False | hypo |
|  | cg13745870 | SPATA12 | False | hypo |
|  | cg07719512 | SLC11A1 | False | hyper |
|  | cg06653796 | LIME1 | True | hyper |
|  | cg06235429 | NDUFV1 | False | hyper |
|  | cg16112945 | ADAMTS13 | False | hyper |
|  | cg10307548 | SOD3 | False | hyper |
|  | cg23124451 | CBX7 | True | hyper |
|  | cg26135325 | LCE3A | False | hyper |

**References**:

1. Yang P, Sun ZF, Krowka MJ, Aubry MC, Bamlet WR, Wampfler JA, et al. Alpha(1)-antitrypsin deficiency carriers, tobacco smoke, chronic obstructive pulmonary disease, and lung cancer risk. Arch Intern Med. 2008;168(10):1097-103. doi: DOI 10.1001/archinte.168.10.1097. PubMed PMID: WOS:000256057000013.

2. Yang P, Allen MS, Aubry MC, Wampfler JA, Marks RS, Edell ES, et al. Clinical features of 5,628 primary lung cancer patients: experience at Mayo Clinic from 1997 to 2003. Chest. 2005;128(1):452-62. Epub 2005/07/09. doi: 10.1378/chest.128.1.452. PubMed PMID: 16002972.

3. Yang P, Wentzlaff KA, Katzmann JA, Marks RS, Allen MS, Lesnick TG, et al. Alpha1-antitrypsin deficiency allele carriers among lung cancer patients. Cancer Epidemiol Biomarkers Prev. 1999;8(5):461-5. PubMed PMID: 10350443.

4. Wang L, Aakre JA, Jiang R, Marks RS, Wu Y, Chen J, et al. Methylation markers for small cell lung cancer in peripheral blood leukocyte DNA. J Thorac Oncol. 2010;5(6):778-85. Epub 2010/04/28. doi: 10.1097/JTO.0b013e3181d6e0b3. PubMed PMID: 20421821; PubMed Central PMCID: PMCPMC3000124.

5. Kilaru V, Barfield RT, Schroeder JW, Smith AK, Conneely KN. MethLAB: a graphical user interface package for the analysis of array-based DNA methylation data. Epigenetics. 2012;7(3):225-9. Epub 2012/03/21. doi: 10.4161/epi.7.3.19284. PubMed PMID: 22430798; PubMed Central PMCID: PMCPMC3335946.

6. Storey JD, Tibshirani R. Statistical significance for genomewide studies. Proc Natl Acad Sci U S A. 2003;100(16):9440-5. Epub 2003/07/29. doi: 10.1073/pnas.1530509100. PubMed PMID: 12883005; PubMed Central PMCID: PMCPMC170937.

7. Huang DW, Sherman BT, Lempicki RA. Systematic and integrative analysis of large gene lists using DAVID bioinformatics resources. Nature Protocols. 2009;4(1):44-57. doi: 10.1038/nprot.2008.211. PubMed PMID: WOS:000265781800006.

8. Shannon P, Markiel A, Ozier O, Baliga NS, Wang JT, Ramage D, et al. Cytoscape: A software environment for integrated models of biomolecular interaction networks. Genome Res. 2003;13(11):2498-504. doi: 10.1101/gr.1239303. PubMed PMID: WOS:000186357000016.

9. Rhodes DR, Kalyana-Sundaram S, Mahavisno V, Varambally R, Yu J, Briggs BB, et al. Oncomine 3.0: genes, pathways, and networks in a collection of 18,000 cancer gene expression profiles. Neoplasia. 2007;9(2):166-80. Epub 2007/03/16. PubMed PMID: 17356713; PubMed Central PMCID: PMCPMC1813932.

10. Reinius LE, Acevedo N, Joerink M, Pershagen G, Dahlen SE, Greco D, et al. Differential DNA Methylation in Purified Human Blood Cells: Implications for Cell Lineage and Studies on Disease Susceptibility. Plos One. 2012;7(7). doi: ARTN e41361 10.1371/journal.pone.0041361. PubMed PMID: WOS:000306806600056.

11. Houseman EA, Accomando WP, Koestler DC, Christensen BC, Marsit CJ, Nelson HH, et al. DNA methylation arrays as surrogate measures of cell mixture distribution. Bmc Bioinformatics. 2012;13. doi: Artn 86 10.1186/1471-2105-13-86. PubMed PMID: WOS:000312891600001.
